# Exploring the Physicochemical, Mechanical, and Photocatalytic Antibacterial Properties of a Methacrylate-Based Dental Material Loaded with ZnO Nanoparticles

**DOI:** 10.3390/ma15145075

**Published:** 2022-07-21

**Authors:** Patricia Comeau, Julia Burgess, Niknaz Malekafzali, Maria Luisa Leite, Aidan Lee, Adriana Manso

**Affiliations:** Department of Oral Health Sciences, Faculty of Dentistry, The University of British Columbia, Vancouver, BC V6T 1Z3, Canada; pcomeau@dentistry.ubc.ca (P.C.); jburge04@student.ubc.ca (J.B.); niknaz@student.ubc.ca (N.M.); mlasl@mail.ubc.ca (M.L.L.); leeaidan@student.ubc.ca (A.L.)

**Keywords:** dental material, methacrylate-based monomers, zinc oxide nanoparticles, photosensitive, photocatalytic, antimicrobial, photodynamic therapy, *Streptococcus mutans*

## Abstract

While resin-based materials meet the many requirements of a restorative material, they lack adequate, long-lasting antimicrobial power. This study investigated a zinc oxide nanoparticle (ZnO NP)-loaded resin-blend (RB) toward a new antimicrobial photodynamic therapy (aPDT)-based approach for managing dental caries. The results confirmed that up to 20 wt% ZnO NPs could be added without compromising the degree of conversion (DC) of the original blend. The DC achieved for the 20 wt% ZnO NP blend has been the highest reported. The effects on flexural strength (FS), shear bond strength to dentin (SBS), water sorption (WS), solubility (SL), and viability of *Streptococcus mutans* under 1.35 J/cm^2^ blue light or dark conditions were limited to ≤20 wt% ZnO NP loading. The addition of up to 20 wt% ZnO NPs had a minimal impact on FS or SBS, while a reduction in the bacteria count was observed. The maximum loading resulted in an increase in SL. Furthermore, 28-day aging in 37 °C water increased the FS for all groups, while it sustained the reduction in bacteria count for the 20 wt% resin blends. Overall, the ZnO NP-loaded resin-based restorative material presents significant potential for use in aPDT.

## 1. Introduction

Dental caries is one of the most prevalent chronic diseases worldwide and affects more than 3.5 billion people [1]. The oral cavity is an ideal environment for biofilm formation, with the bacterial composition of these biofilms relatively stable and protected from conventional biofilm-removing forces applied in the mouth [2]. The cariogenic biofilms built on tooth surfaces can lead to dental caries and cause irreversible inflammation, infection and necrosis of the pulp tissue, and, eventually, tooth loss [3,4]. While mechanical removal of oral biofilms is the most effective way to prevent dental caries, the challenges associated with the patients’ compliance to an oral hygiene routine, combined with the required dexterity to properly perform it in all critical areas, are key determinants of its success. In order to address the need for a long-lasting dental caries management strategy, many researchers have focused on the development of novel dental materials for the control and management of caries, thus limiting their development, progression, and/or recurrence.

Restorative materials used to repair defects from carious lesions include dental amalgam, dental cements (e.g., glass-ionomer), and polymeric resin-based materials [5]. Due to their superior aesthetics and bonding ability, resin-based dental materials have predominantly replaced amalgam restorations in direct restorative procedures for posterior teeth. However, when compared to other restorative alternatives, resin composites tend to attract more biofilm [6,7]. Furthermore, the polymerization shrinkage occurring at the margins may create a gap at the tooth–resin interface, thus becoming more susceptible to secondary caries [8,9]. The incorporation of antimicrobial additives into the resin has been shown to improve the action against caries-specific microbiological species [10,11,12,13]. In addition, for long-term clinical applications, these additives must sustain antimicrobial properties while not acting detrimentally on the mechanical, physical, and/or chemical properties of the dental material.

Metallic nanoparticles (NPs) are one type of additive that have earned a significant amount of interest for their potential to improve the antibacterial properties of resin-based dental materials [2,5]. Nanoparticles have a particularly high antimicrobial activity at low concentrations due to their quick infiltration of the oral biofilm and bacteria cell walls as well as their large surface area-to-mass ratio [2,14,15,16,17]. However, while silver nanoparticles are one of the most common metallic nanoparticles added to materials to improve antibacterial potential due to the reduction reaction of silver ions to metallic silver, the resin becomes significantly discolored with a dark appearance [18,19]. As such, they can compromise the aesthetics and may limit their application in more anterior areas of the mouth.

More recently, zinc oxide nanoparticles (ZnO NPs) have been of increasing interest as they do not lead to significant discoloration and are both nontoxic and biocompatible [20]. Several studies have shown the antimicrobial activity of aqueous mixtures of ZnO NPs against Gram-positive [21,22,23] and Gram-negative bacterial species [21,22,23,24,25], with some also displaying antifungal activity [26,27,28]. The inclusion of these ZnO NPs in dental materials has also shown improved antibacterial potential against cariogenic bacteria [29,30,31,32,33,34]. However, challenging this approach is the evidence that the inclusion of ZnO NPs alone in resin-based restoratives have minimal antibacterial effects against multi-species biofilms [35] and may also not be suitable for long-lasting clinical applications [32,35]. Thus, as most resin-based restorations are expected to survive for at least 10 years, the key to restorative dentistry success may now involve unlocking a more substantial and long-lasting potential for ZnO NP antimicrobial action. Antimicrobial photodynamic therapy (aPDT), which has been of increasing interest in many medical fields, is a possible alternative to be explored in the development of novel dental materials containing ZnO NPs.

In aPDT, the application of light toward a photosensitive compound in the presence of oxygen produces reactive oxygen species (ROS), which ultimately cause the death of pathogenic microorganisms including cariogenic bacteria such as *Streptococcus mutans* (*S. mutans*) [36]. While traditional photosensitive compounds used in aPDT are organic chemicals, the semiconductive nature of ZnO NPs make them a useful (and more cost-effective) alternative for application in aPDT. When ZnO NPs are irradiated with ultra-violet (UV) or visible light, electron(s) move from the valence band to the conduction band and electron–hole pair(s) are formed [37]. Within the conduction band, electrons then reduce oxygen to produce O_2_*^−^, while the electron-hole remaining in the valence band reacts with water to generate HO*. These two forms of ROS produced upon light irradiation of the ZnO NPs have a bacteriostatic effect [37,38]. In addition, in an acidic environment or microenvironment such as that found in caries-prone patients at specific sites in the mouth, ZnO NPs dissociate to produce divalent zinc ions, which themselves have been shown to be antimicrobial [39]. While most studies have applied UV light to induce a photodynamic response [40,41,42,43,44], there has also been some success in applying light to ZnO NPs via blue light-emitting diodes (LEDs) [45]. This latter approach is particularly appealing due to the low operation cost, ease of use, and compact size and portability of blue LED sources in dentistry; these are readily available in all dental practices as a portable light curing device.

Considering the broad antimicrobial effect of ZnO NPs when incorporated in resin-based dental materials, combined with the potential of these NPs in an aPDT approach, it is clear that further investigations are needed to improve the scientific understanding of such strategies, particularly concerning their long-term effectiveness. This greater understanding will then be beneficial when we put this novel technology in perspective toward the clinical application for dental caries management. Therefore, the primary objective of this study was to evaluate the physical, mechanical, and chemical properties of an experimental methacrylate-based resin loaded with different concentrations of ZnO NPs. In order to develop antimicrobial dental resin blends that are clinically relevant, the incorporation of ZnO NPs must not detrimentally impact the degree of conversion (DC), flexural strength (FS), shear bond strength (SBS), or water sorption/solubility (WS/SL) of the resin. We hypothesized that beyond a maximum ZnO NP concentration, the DC will significantly decrease, but by limiting subsequent loading to below this maximum, the aforementioned resin properties will not be detrimentally impacted. The secondary objective was to assess the antimicrobial potential of the experimental resin blend loaded with ZnO NPs, and the effects of an aPDT approach on this novel dental biomaterial against cariogenic bacteria following artificial aging and the application of light from a blue LED-based device.

## 2. Materials and Methods

### 2.1. Experimental Methacrylate-Based Resin Blend Loaded with ZnO NPs

A methacrylate-based experimental resin blend (RB) was first formulated using tetraethylene-glycol-dimethacrylate (TEEGDMA, 30 wt%), ethoxylated (4) bisphenol A dimethacrylate (Bis-EMA, 50 wt%), 2-hydroxyethyl methacrylate (HEMA, 14 wt%), ethanol (EtOH, 4 wt%), and a photoinitiator system consisting of camphorquinone (CQ, 0.66 wt%) and ethyl 4-(dimethylamino) benzoate (Amino, 1.34 wt%). The freshly mixed RB was then loaded with 1, 5, 10, 15, 20, 25, 30, 35, or 40 wt% of ZnO NPs (50–150 nm, 99.9% pure; M K Impex Corp., Mississauga, ON, Canada). The unloaded RB (0% ZnO NPs) served as the study control for all tests.

TEEGDMA, Bis-EMA, and HEMA were purchased from Scientific Polymer Products Inc. (Ontario, NY, USA), while CQ and Amino were purchased from Sigma Aldrich, Inc. (St. Louis, MO, USA).

### 2.2. Initial Screening of Different ZnO NP Concentrations Added to Resin Blend

All nine experimental blends and the control (0%) were analyzed on a Fourier Transform Infrared (FTIR) spectrometer (IRPrestige-21, Shimadzu^TM^, Kyoto, Japan) for DC at 30 or 60 s curing times using a Bluephase^®^ light curing unit (LCU, Ivoclar Vivadent^®^, Schaan, Liechtenstein) operating at ~1300 mW/cm^2^. Spectra were collected over a range of 400–4000 cm^−1^ at a resolution of 4.0 cm^−1^ with 64 scans (*n* = 3). The DC was calculated using Equation (1) and absorbance (Abs) was set at 1608 cm^−1^ and 1636 cm^−1^:

(1) $$\%\;\mathrm{DC}=(1\;-\;\left[\frac{{\mathrm{cured}\;\mathrm{sample}\;(\mathrm{Abs}\;(1636\;\mathrm{cm}}^{-1}{)/\mathrm{Abs}\;(1608\;\mathrm{cm}}^{-1}))}{{\mathrm{uncured}\;\mathrm{sample}\;(\mathrm{Abs}\;(1636\;\mathrm{cm}}^{-1}{)/\mathrm{Abs}\;(1608\;\mathrm{cm}}^{-1}))}\right])\times 100\%$$

In addition, using polyvinyl siloxane (PVS) molds (6 mm diameter × 1 mm thickness), resin disks were fabricated with each blend and photopolymerized for 60 s on each side with a Valo^TM^ LCU (Ultradent Products, Inc., South Jordan, UT, USA) at 830 mW/cm^2^. Altogether, the DC analysis and visual assessment of the physical integrity of the disks served as an early screening of the ZnO NPs-loaded experimental RBs. Next, three optimal formulations (5, 10, and 20 wt% ZnO NPs) were selected and further characterized against the unloaded control blend (0 wt%).

### 2.3. Water Sorption and Solubility (WS and SL)

Resin disks (6 mm diameter × 1 mm thickness) were fabricated using PVS molds and light-cured as previously described (*n* = 6). The mass (*m*_1_) and volume (*V*) of the resin disks for each experimental group were then recorded before adding them to 37 °C MQ^®^ ultrapure water for 7 days. At regular daily intervals, each disk was dried, weighed, and returned to the 37 °C ultrapure water. After 7 days, the disks were weighed (*m*_2_) and then stored for drying in a 37 °C oven until a constant weight was reached (*m*_3_). The water sorption (*WS*) and solubility (*SL*) were calculated following Equations (2) and (3), respectively [46].

(2) $$WS=\frac{\left(m_{2}-m_{3}\right)}{V}$$

(3) $$SL=\frac{\left(m_{1}-m_{3}\right)}{V}$$

### 2.4. Flexural Strength (FS)

The methacrylate-based dental resin blends, loaded with either 0, 5, 10, or 20 wt% ZnO NPs, were tested for flexural strength following two storage times (24 h or 28 d). First, a Teflon matrix was used to produce the FS bars with a length of 25 mm and a 2 mm × 2 mm cross-section (*n* = 12). The samples were light-cured in three different segments along the length of the top and bottom surfaces (60 s, each segment) with a LED curing unit (~830 mW/cm^2^; Valo^TM^). The FS bars were then stored in 37 °C MQ^®^ ultrapure water for 24 h (“Fresh”) or 28 d incubation times (“Aged”). The samples were tested on a Universal Testing Machine (Shimadzu^TM^, Kyoto, Japan) at a 1 mm/min cross-head speed in accordance with the ISO 10477 standard. 

### 2.5. Shear Bond Strength to Dentin (SBS)

Extracted sound human molars (REB-H14-02189) were first sectioned coronally and polished with 600-grit SiC sandpaper to expose the mid-coronal dentin. Each surface was then etched (15 s, phosphoric acid), rinsed, primed (15 s active application of 3M ESPE Adper^TM^ Scotchbond^TM^ Multi-Purpose primer), bonded with one experimental RB (0, 5, 10, 20 wt% ZnO NPs) or the commercial control (3M ESPE Adper^TM^ Scotchbond^TM^ MP adhesive), and light-cured (20 s) with the Valo^TM^ LCU at 830 mW/cm^2^. An Ultradent^TM^ jig was used to create a composite build-up (Estekute Quick Tokuyama Dental Corp., Taito City, Tokyo, Japan) and light-cured for 40 s. After 24 h (“Fresh”) or 28 d (“Aged”) oof storage in 37 °C MQ^®^ ultrapure water, the specimens were tested in shear mode (BISCO^TM^ tester) at 0.5 mm/min (*n* = 12). Shear bond strength (SBS) was calculated by the division of the maximum stress supported (N) before failure and the dentin/specimen bonded area (mm^2^).

### 2.6. Bacterial Strain and Growth Conditions

A standard strain of *S. mutans,* UA159 from the American Type Culture Collection (ATCC 700610; Rockville, MD, USA), was used to form the single-species biofilms. Stock cultures were maintained at −80 °C, reactivated onto 5% Sheep Blood Agar plates (BBL^TM^, Becton, Dickinson and Company, Sparks, MD, USA) and incubated in a 5% CO_2_ incubator (Isotemp CO_2_ incubator, Thermo Fisher Scientific, Marietta, OH, USA) at 37 °C for 48 h. After that, for the formation of the pre-inoculum, the bacteria were individually reactivated by transferring single colonies (10–12) to a tube containing 5 mL of brain-heart infusion (BHI) broth culture medium (BD BBL^TM^, Becton, Dickinson and Company, Sparks, MD, USA) supplemented with 1% glucose and kept overnight in an incubator (5% CO_2_, 37 °C). The inoculum started from an absorbance of 0.08–0.10 as read at an optical density (OD) of 600 nm (BioTek^®^, Winnoski, VT, USA), corresponding to 1.5 × 10^8^ colony forming units (CFU)/mL. The inoculum was then diluted with BHI broth to obtain a final *S. mutans* concentration of 1 × 10^6^ CFU/mL.

### 2.7. S. mutans Biofilm Formation and Treatments

The evaluation of antimicrobial activity of the experimental RBs were performed by producing additional disks (6 mm diameter × 1 mm thickness) of the blends (0, 5, 10, 20 wt% ZnO NPs) (*n* = 9) as previously reported, using PVS molds and the Valo^TM^ LCU (830 mW/cm^2^). Immediately following disk fabrication (“Fresh”) or after 28 d of aging in 37 °C water (“Aged”), the disks were autoclaved at 121 °C for 30 min. The disks were then incubated with 0.5 mL of the diluted *S. mutans* inoculum at 1 × 10^6^ CFU/mL in 24-well plates (Thermo Fisher Scientific, Rochester, NY, USA) at 37 °C (5% CO_2_) (Isotemp CO_2_ incubator, Thermo Fisher Scientific, Marietta, OH, USA). The biofilms were left undisturbed for 6 h to allow for early-stage biofilm formation at 37 °C under microaerophilic conditions. After 6 h incubation time, the samples were washed with 1 mL of sterile phosphate buffered saline (PBS) and one of the following treatments was performed: (1) blue-light treatment (“Light”) for 60 s at a distance of ~18 mm from the sample or (2) no light treatment (“Dark”). The light treatment was performed using a customized light device, which could irradiate all specimens simultaneously. The device had one blue LED (λ = 440–460 nm, ~23 mW/cm^2^ per LED) centered above each well of the 24-well plate. In this case, the 60 s light exposure is equivalent to each sample receiving ~1.35 J/cm^2^ of irradiant energy.

### 2.8. S. mutans CFU Count Response Assay

After treatment, the resin disks were transferred to pre-labelled microcentrifuge tubes containing 1 mL sterile PBS and sonicated for 30 s at amplitude 20 (QSonica sonicators, Newton, CT, USA). Serial dilutions, by a factor of 10 until a final serial dilution of 10^−7^ was obtained, were performed in sterile PBS and plated onto blood agar plates. Plates were incubated for 48 h and colonies were counted to obtain the bacterial viability measured in CFU/mL. The CFU concentration was calculated using Equation (4). All tests were performed in triplicate and in three independent runs (i.e., *n* = 3 × 3 or total *n* = 9).

CFU/mL = [(# of colonies) × (1/dilution factor) × 1000 µL/mL]/(volume plated)(4)

### 2.9. Data Analysis

Sample preparation was randomized with multiple resin blend batches prepared for each optimal formulation and control blend, and multiple light-cured samples at each ZnO wt% were prepared at any given fabrication time. In addition, for select characterization (e.g., FS, SBS, antibacterial), multiple runs were conducted. Furthermore, prior to any testing, the light-cured samples were independently inspected for quality, ensuring that no samples with visible bubbles or other defects were tested. All statistical analysis was conducted using the IBM^®^ SPSS^®^ Statistics v28 software package (Armonk, New York, USA). The data were analyzed with a multi-factor univariate general linear model and post-hoc Tukey tests (α = 0.05). Data were presented as the mean ± one standard deviation (SD). Statistical significance was set as *p* < 0.05.

## 3. Results

### 3.1. Degree of Conversion (DC)

**Figure 1:**
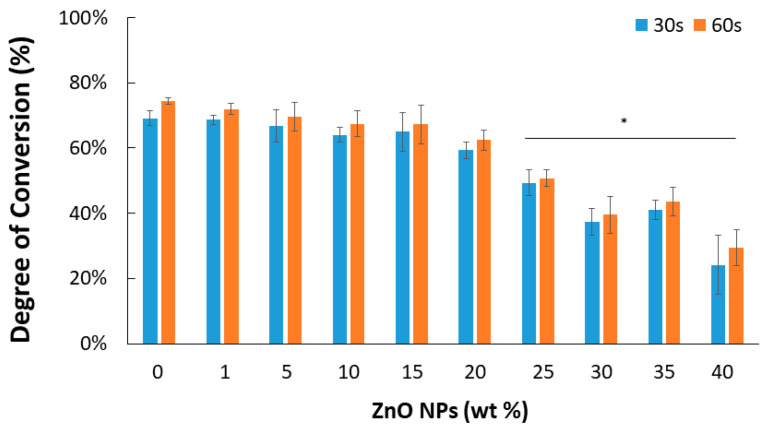
The degree of conversion (DC) for the methacrylate-based resin blends. * Indicates a statistically significant decrease from the control resin blend (0 wt% ZnO NPs) (* *p* < 0.05; *n* = 3).

Results for the FTIR analysis confirmed that both the concentration of ZnO NPs and light-curing time detectably impacted the DC for the experimental methacrylate-based RBs (Figure 1; *p* < 0.001 and *p* = 0.007, respectively). Independent of light-curing time (30 or 60 s), loading with 25 wt% (or more) ZnO NPs significantly reduced the DC. Representative spectra matching each light-curing time and ZnO NP wt% are shown in the Supplementary Materials (Figure S1).

**Figure 2:**
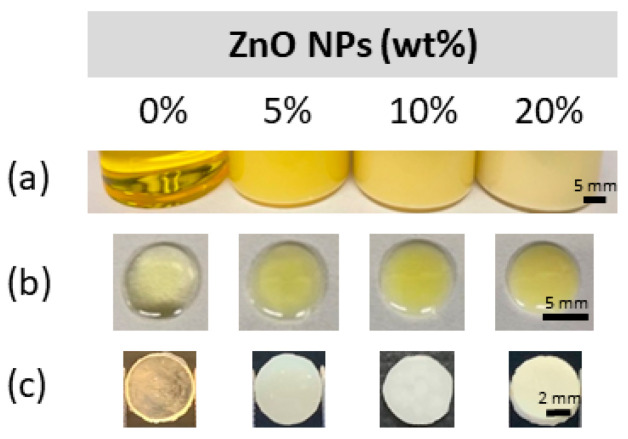
(**a**) Resin blend within storage vial. (**b**) Drop of resin blend on the glass slide prior to light curing. (**c**) Resin disk following fabrication.

Meanwhile, in a subsequent experiment, adding up to 20 wt% ZnO NPs was found to have no impact on the structural stability of the RB samples following our standard fabrication protocol with 60 s light curing on both the top and bottom surfaces. A representative image of each optimal blend (0, 5, 10, and 20 wt% ZnO NPs) prior to and post-light curing is shown in Figure 2.

### 3.2. Water Sorption (WS) and Solubility (SL)

**Figure 3:**
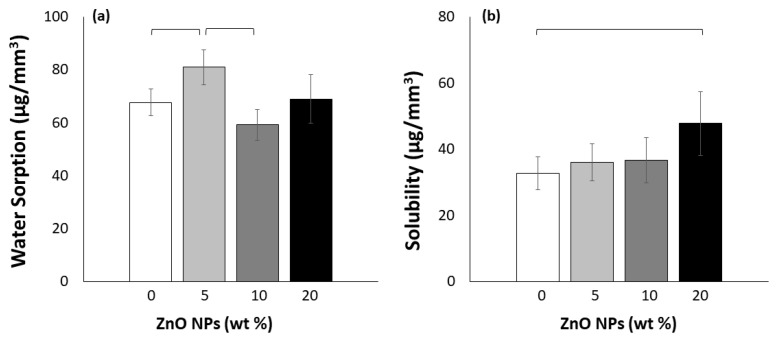
(**a**) Water sorption and (**b**) solubility of the methacrylate-based resin blends. Horizontal bars indicate a significant difference (*p* < 0.05; *n* = 6).

The incorporation of ZnO NPs detectably impacted the WS and SL of the experimental RB (Figure 3; *p* < 0.001 and *p* = 0.013, respectively). The 5 wt% ZnO NP-loaded RB resulted in the highest WS compared to the unloaded RB (*p* = 0.020) and 10 wt% ZnO NP-loaded RBs (*p* < 0.001). The SL analysis showed that only the 20 wt% ZnO NP-loaded RB had a significantly higher SL than the unloaded RB (*p* = 0.010).

### 3.3. Flexural Strength (FS)

**Figure 4:**
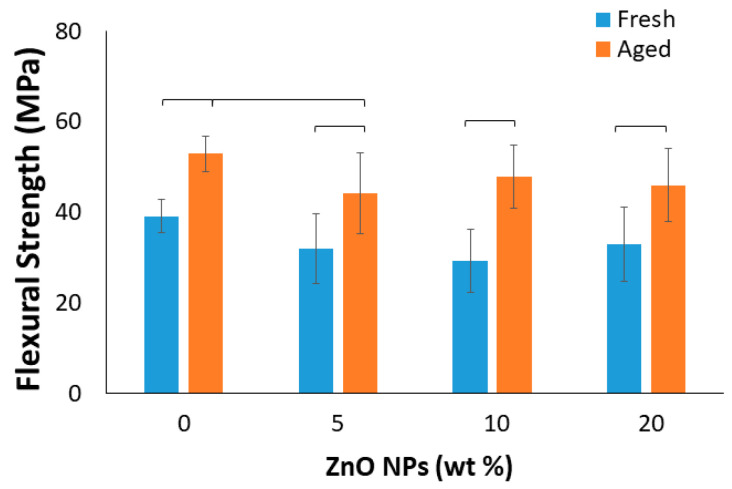
The flexural strength of the methacrylate-based resin blends. Horizontal bars indicate a significant difference (*p* < 0.05; *n* = 12).

The results demonstrated that both the ZnO NP concentration and 28 d aging in 37 °C water detectably impacted the FS of the experimental methacrylate-based RBs (Figure 4; *p* < 0.001 for both). Independent of the ZnO NP concentration, the 28 d aging in 37 °C water significantly increased the FS of the RBs (*p* ≤ 0.010 for all). Meanwhile, following 28 d of artificial aging, only the 5 wt% ZnO NP-loaded RB presented with a statistically lower FS than the unloaded and aged RB (*p* = 0.044).

### 3.4. Shear Bond Strength to Dentin (SBS)

**Figure 5:**
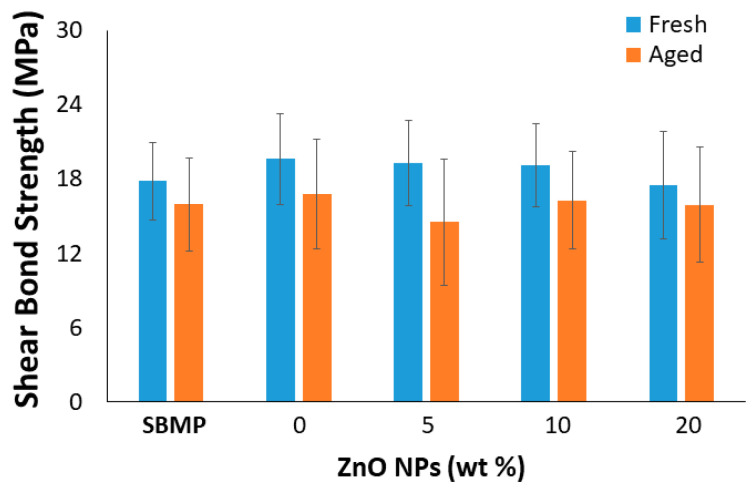
The shear bond strength (SBS) of the methacrylate-based resin blends (*n* = 12).

The results for the SBS to dentin of the experimental resin blends were consistent with the commercial control of Scotchbond^TM^ Multi-purpose (SBMP), with no significant differences among them for both the fresh and 28 d aged sample sets (Figure 5). While the two-factor general lineal model indicated a difference between storage time when data from all experimental groups were combined (*p* < 0.05), there was no detectable impact of the ZnO NP concentration on the SBS of the experimental RBs to dentin.

### 3.5. S. mutans CFU/mL Count Response Assay

**Figure 6:**
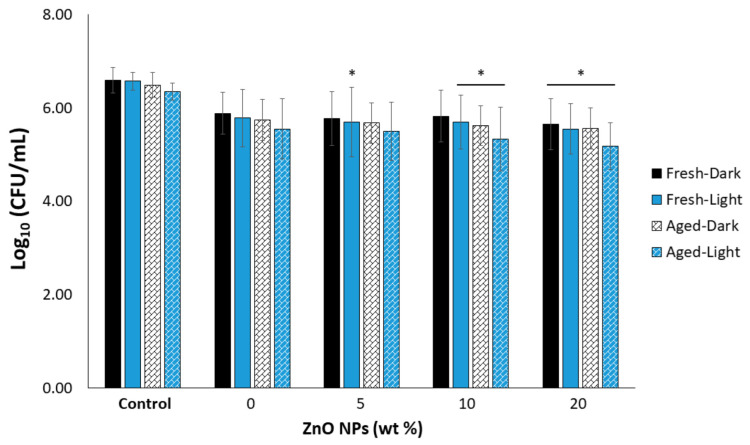
The CFU/mL count as a function of ZnO NP concentration, aging in 37 °C water, and application of light. * Indicates a detected decrease from the *S. mutans* control under similar aging and light conditions (* *p* < 0.05; *n* = 9).

The results demonstrated that higher concentrations (10 and 20 wt%) of ZnO NPs had a positive impact on the antimicrobial potential of the experimental RBs (Figure 6). Aging of the specimens for 28 d in 37 °C water prior to forming the biofilm presented as the most significant independent variable overall (*p* = 0.025). The specimens with 20 wt% ZnO NPs incorporated into the RB displayed a significantly reduced CFU/mL count for all experimental groups compared to the *S. mutans*-only control. The statistical analysis was presented as follows: “Fresh-Dark” group (*p* = 0.026), “Aged-Dark” group (*p* = 0.021), “Fresh-Light” group (*p* = 0.006), and “Aged-Light” group (*p* < 0.001). Furthermore, all experimental groups for the 10 wt% ZnO NP-loaded RB, except for the “Aged-Dark” condition, showed a statistically significant decrease in the CFU/mL count compared to the control (*p* < 0.05).

## 4. Discussion

Contemporary nanotechnology has shown significant potential in both preventive and restorative dentistry, which has led to the synthesis of a new class of biomaterials. While there is strong antibacterial evidence for incorporating ZnO NPs into dental materials [30,31,32,34,35], there is conflicting evidence about whether its antibacterial promise can be maintained over time, what bacteria it is most effective against, and what the optimal concentration is to produce an antimicrobial response. Antimicrobial photodynamic therapy (aPDT) is an approach relatively new to restorative dentistry, which may significantly improve the long-term efficacy of the proposed antimicrobial dental material. Specific light wavelengths applied to ZnO NPs can trigger photochemical reactions, leading to the generation of ROS that can cause damage to the organelles, cell membranes, and cell nuclei of bacteria, enhancing its antimicrobial potential [47]. ZnO NPs demonstrate several peaks in fluorescence including a strong blue emission peak at 445 nm [48], and can act as a photosensitizer in aPDT applications. By adding ZnO NPs into our experimental resins, the goal was to maintain the photosensitizing ability over a certain aging time without impacting other relevant properties. Therefore, ZnO NPs were loaded into experimental dental RBs and their physicochemical, mechanical, and antimicrobial properties as a function of ZnO NP concentration and aging were evaluated.

Initially, the experimental methacrylate-based resin blend was loaded with nine different concentrations of ZnO NPs in the range of 1 to 40 wt%. The aim was to select the ideal concentrations that were not detrimental to the experimental resin blend regarding the conversion of the methacrylate monomers into polymeric chains (i.e., degree of conversion [DC]). It was observed that, independent of the light curing time applied (30 or 60 s), the DC of the experimental RBs significantly decreased upon the addition of ≥25 wt% ZnO NPs. However, the RBs containing ≤20 wt% ZnO NPs had a DC similar to that of the unloaded RB. This has been corroborated by other studies including a study by Collares et al. where adding 30 or 40 wt% of 40 nm ZnO NPs to a slightly different methacrylate-based monomer significantly decreased the DC [34]. However, in our study, the DC of formulations containing 20 wt% was notably higher than these earlier studies as it presented with a DC above 55%, a DC considered by some researchers to be the minimal required value for clinical success [49]. As the conversion of monomers into polymers has been found to directly correlate to the observed physical, chemical, and mechanical properties of resins [50,51], it is important that this new dental material be properly screened for DC before further mechanical and microbiological tests. In addition, 1-mm-thick disk specimens containing ≤20 wt% ZnO NPs were successfully light-cured and fabricated, thus confirming the feasibility of such concentrations for the next experimental steps. Due to the DC analysis and observations from the specimen preparation, it is plausible that above a given concentration, the ZnO NPs would detrimentally affect light transmission and photoinitiator action due to light scattering and attenuation, which could be further exasperated by the formation of NP agglomerates [52]. As a result, the optimal concentrations were determined to be 5, 10, and 20 wt% ZnO NPs, with the unloaded RB (0 wt%) serving as a study control.

An ideal restorative material should be practically insoluble, with both high chemical and thermal stability; however, in an oral environment, most materials will absorb and release some water and chemicals, respectively [46]. In this study, only the 5 wt% ZnO NP-loaded RB presented a significant increase in WS compared to the unloaded RB, while for the SL analysis, only the 20 wt% ZnO NP-loaded RB incurred a significant increase in SL. Furthermore, it is important to note that the change observed in the 20 wt% ZnO NP-loaded RB SL test was more considerable than for that of the WS test, with an increase in magnitude of 42% compared to the control. As there was no tuning of the NP surface to improve the particle–RB interaction, it is plausible that the interface interactions between the ZnO NPs and the RB matrix were insignificant in this study. This, in turn, would encourage any uncured monomer to readily leach out of the RB, or water to influx in when the specimens were in an aqueous solution [53].

We observed that the ZnO concentration had a minimal impact on the FS and SBS tests of the fresh sample (i.e., 24 h storage). While previous studies have observed similar FS and SBS of dental sealants [54], resin composites [32], and dental adhesives [30,31] loaded with ZnO NPs, most of these studies were limited to ≤10 wt% NPs. In addition, to our knowledge, this is the first study to analyze the FS and SBS properties of a methacrylate-based RB loaded with ZnO NPs after 28 d of aging in 37 °C water. Interestingly, after 28 d of storage in water, only the 5 wt% ZnO NP-loaded RB displayed detectably decreased FS compared to the control (0%). This finding aligns with the WS finding, where the 5 wt% concentration was also the only one presenting an increase in WS compared to the unloaded RB. This led us to speculate that the increased water absorption into the 5 wt% ZnO NP-loaded RB likely weakened the resin matrix under FS testing. In addition, a significant increase in FS was demonstrated after 28 d of aging (in 37 °C water) for all of the formulations tested. It has previously been argued that enhanced FS following aging could be due to temperature-derived mobility of polymeric chains and consequently entrapped radical engagement so that the DC increased [55,56,57]. In contrast to the FS results, the 28 d aging did not result in a similar change to the SBS. However, it is important to put into perspective that the SBS test only evaluates the effects of the ZnO NP loading on the bonding ability of the experimental RBs to dentin. The findings of this study show promise with regard to the SBS stability and overall performance, particularly because they presented results comparable to our commercial control; this commercial product has been on the market for over 20 years and has been extensively evaluated by several research groups. Indeed, significant drops in SBS over time are commonly reported in the literature [58,59], even for materials regarded as the “gold standard” such as SBMP [60,61]. Our experimental RBs did not display significant reductions in SBS to dentin after aging, and added to the fact that the 24 h SBS results are comparable to the commercial control, this new ZnO NP-loaded RB shows significant promise in adhesive restorative dentistry.

*Streptococcus mutans* (*S. mutans*) is one of the most common microorganisms used in the screening of dental materials for antimicrobial properties against cariogenic biofilms. This is due to their direct involvement in the early attachment and formation of cariogenic biofilms [62,63]. In this study, a single species microbiological evaluation model was used to assess the impact of ZnO NP concentration (0, 5, 10, 20 wt%), aging (fresh or 28 d), and dark or blue light application (1.35 J/cm^2^) on 6 h *S. mutans* biofilms. While no treatment groups in this study resulted in a 3 log_10_ reduction, which has been considered biologically relevant by the American Society for Microbiology and CLSI standard [64], a few groups did produce a small, but detectable drop in CFU/mL count compared to the bacteria-only control. For instance, all 20 wt% ZnO NP groups and three of the 10 wt% ZnO NP groups led to a statistically significant 0.8–1.2 log_10_ reduction compared to the bacteria-only control.

The antimicrobial potential of ZnO against cariogenic bacteria has already been demonstrated in both aqueous mixtures and contained within various dental materials [30,31,32,35,54]. However, there is very little consistency amongst such studies due to the differences in materials and formulations, the nature of ZnO NPs (including particle size and shape), and the antimicrobial testing methods and approaches involved, thus making any direct comparisons challenging. For instance, our study is the first to use this particular resin blend formulation together with 50–150 nm ZnO NPs. It is even more challenging to compare ZnO NP-loaded dental formulations when very few publications consider the response of such blends to an aqueous environment, specifically with regard to any confirmed correlation between water sorption, solubility, and/or ion release with the antimicrobial response. Such information would considerably improve the knowledge around antimicrobial response, and, for example, whether it is a more active or passive process. A commonly reported characterization parameter for polymeric dental materials is, however, the DC, and our experimental RBs showed significant promise by obtaining a DC greater than 55% with as much as 20 wt% ZnO NPs included in the RB. In dental resins presenting lower DC, the long-term stability of the material would be expected to be in greater jeopardy, but it could also result in greater zinc ion release (with some dependence on light irradiation). Thus, while ion release was not measured in this study, the findings of previous studies indicated that a greater antimicrobial response of the ZnO-added adhesive [31] and resin composite [32] is likely due, at least in part, to the corresponding DC being notably much lower than 55%. Future investigation will also consider measuring the release of Zn^2+^ from our material system and correlating it with bacteria assays in order to improve the understanding of the mechanism(s) involved in producing an agonistic antibacterial response. Furthermore, while 6 h is an acceptable early-stage period of biofilm growth, there is some evidence that concentration-dependent antimicrobial responses are more evident when longer times for biofilm growth are used [32].

Of great significance in this study is that the reduced CFU/mL count observed by most treatments on the 10 and 20 wt% ZnO NP-loaded RBs was sustained after 28 d of aging. The few studies that have evaluated the antimicrobial potential of the ZnO NP-loaded formulations have observed a decrease in such properties after aging [32,35]. While applying light to the specimens did not further increase the antimicrobial response, it is important to emphasize that this is the first evaluation of our ZnO NP-loaded RB for an aPDT approach. As such, the light protocol utilized a relatively low energy dose of only 1.35 J/cm^2^ from blue LEDs. This energy is low when compared to the scarce literature highlighting the photocatalytic behavior of ZnO NPs, but reflects a 1-min light treatment, a feasible approach for clinical or at home therapies. A study by Wang et al. (2019) evaluated the response of *S. mutans* to aqueous suspensions of various metallic NPs including ZnO NPs using a slightly higher blue LED energy dose of 10 to 51 J/cm^2^ [45]. Interestingly, this higher energy dose was not found to reduce the CFU/mL count compared to the non-irradiated samples, despite more Zn^2+^ being released following irradiation, and it was then suggested that ion release is not the predominant mechanism in creating an antimicrobial response [45]. As such, the aqueous stability of our RB when loaded with ZnO along with the higher DC are not believed to be limiting factors in the antimicrobial response of the new material. From the literature it is known that the smallest-sized and specifically-shaped ZnO NPs [41,65,66], higher light energy [67,68,69], and certain biofilm properties such as age [70,71] are more likely to reveal the antimicrobial properties, though the extent of the impact for such factors is likely dependent on the type of photosensitizer (PS). For example, a longer biofilm growth period allows for the formation of a mature biofilm that creates a cariogenic environment with low pH (pH < 5.5) [72], and due to this lower pH, higher ZnO dissociation would occur [73]. Future work will also consider using smaller ZnO NPs, higher light energy, and studies using longer biofilm growth.

## 5. Conclusions

The feasibility of incorporating photosensitive ZnO NPs into an experimental methacrylate resin blend without detrimentally impacting most of the physicochemical, mechanical, and adhesive properties was demonstrated in this study. The maximum optimal loading of ZnO NPs based on DC and 1-mm thick sample fabrication was found to be 20 wt%. Interestingly, the addition of up to 20 wt% ZnO NPs maintained the DC above 55%—a magnitude not previously reached in other ZnO-loaded resin blends and is significant for restorative longevity. Of interest also in this study was that aging both increased flexural strength and maintained the antimicrobial properties of the specimens, independent of the ZnO concentration. Future studies will seek to optimize the resin chemistry and blue light parameters toward producing a stronger aPDT response.

## Data Availability

The authors confirm that the data supporting the findings of this study are available within the article and may be further provided upon reasonable request to the corresponding author.

## Supplementary Materials

The following supporting information can be downloaded at: https://www.mdpi.com/article/10.3390/ma15145075/s1, Figure S1: Representative FTIR spectra for each RB.
