# Supplementary material for: Exploring the Physicochemical, Mechanical, and Photocatalytic Antibacterial Properties of a Methacrylate-Based Dental Material Loaded with ZnO Nanoparticles
Source: Materials (Basel). 2022 Jul 21;15(14):5075. doi: 10.3390/ma15145075 (PMC9319981; doi:10.3390/ma15145075)
Supplement: Supplementary file 1 [file materials-15-05075-s001.zip › materials-1784594-supplementary.pdf]

*materials*

MDPI

*Article*

# Exploring the Physicochemical, Mechanical, and Photocatalytic Antibacterial Properties of a Methacrylate-based Dental Material Loaded with ZnO Nanoparticles

**Patricia Comeau [1], Julia Burgess [1], Niknaz Malekafzali [1], Maria Luisa Leite [1], Aidan Lee [1], and Adriana Manso [1,*]**

[1] Department of Oral Health Sciences, Faculty of Dentistry, The University of British Columbia, Vancouver, BC, Canada, V6T 1Z3

* Correspondence: amanso@dentistry.ubc.ca; Tel.:1-604-822-0383

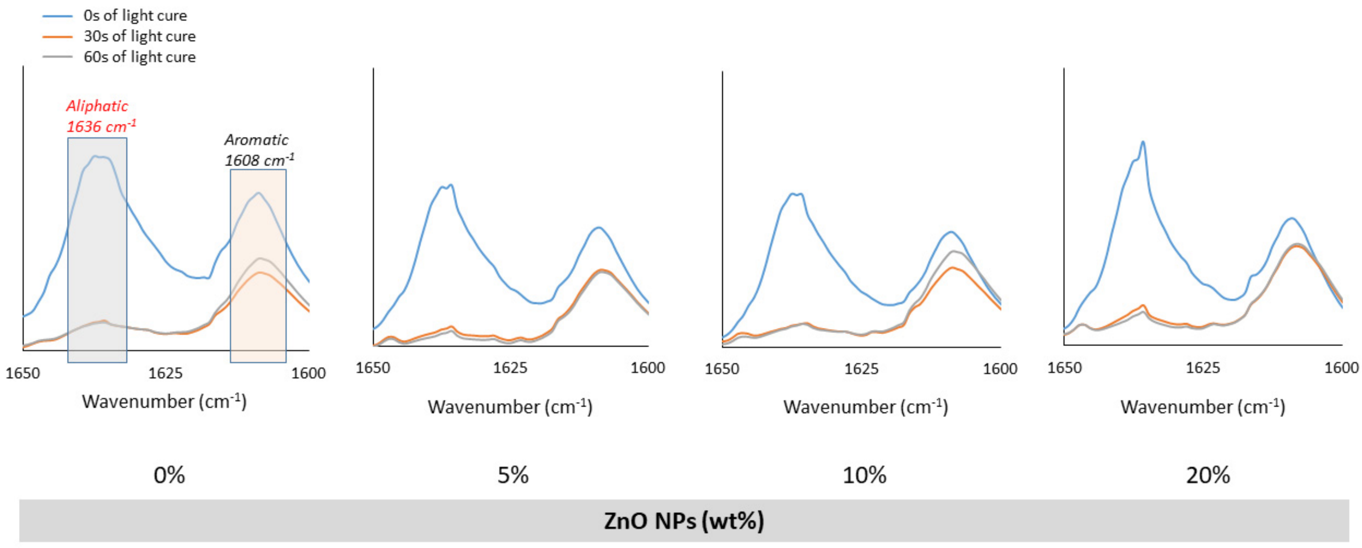


**Figure S1.** Representative FTIR Spectra for each RB.
